# Intermittent hemodialysis: a review of the top antimicrobial stewardship practices to be employed

**DOI:** 10.1017/ash.2023.525

**Published:** 2024-01-11

**Authors:** Nour Shamas, Faryal Khamis, Khalid Eljaaly, Zaher Al Salmi, Maher Al Bahrani

**Affiliations:** 1 Infection Prevention and Control Department, Ministry of National Guard Health Affairs, Riyadh, Saudi Arabia; 2 Division of Infectious Diseases, Department of Internal Medicine, Royal Hospital, Muscat, Oman; 3 Department of Pharmacy, King Abdulaziz University, Jeddah, Saudi Arabia; 4 Department of Pharmaceutical Care, Royal Hospital, Muscat, Oman; 5 Department of Anesthesia and Critical Care, Royal Hospital, Muscat, Oman

## Abstract

The vulnerability of patients on hemodialysis (HD) to infections is evident by their increased susceptibility to infections in general and to resistant organisms in particular. Unnecessary, inappropriate, or suboptimal antimicrobial prescribing is common in dialysis units. This underscores the need for dedicated antimicrobial stewardship (AMS) interventions that can be implemented both in the inpatient and outpatient settings. In this review, we provide a comprehensive approach for clinicians with the most updated coordinated AMS principles in HD setting in six areas: prevention, diagnosis, treatment, education and empowerment, monitoring, and research.

## Introduction

Renal failure patients on hemodialysis (HD) are at great risk of developing infections, leading to high rates of morbidity and mortality in this patient population. The cumulative annual incidence of infection-requiring hospitalization in patients with end-stage renal failure was reported to be 31% in adults, where catheter-related bloodstream infections (CRBSIs) account for most of such incidents.^1^ Furthermore, dialysis patients are prone to HAIs during treatment or hospitalization. Frequent antimicrobial exposure in these patients can lead to the emergence and dissemination of multidrug-resistant (MDR) organisms, hence, arising the need for dedicated antimicrobial stewardship (AMS) interventions that reduce this antimicrobial burden. The evidence on optimal AMS interventions in HD is scarce, and the recently published antimicrobial resistance (AMR) research agenda specifically identifies the need to evaluate the current situation and interventions in vulnerable patient populations, which includes HD.^2^ A recent whitepaper by Apata et al highlighted various approaches to AMS initiatives for the outpatient HD setting that can also be extrapolated to the inpatient setting.^3^ In this article, we propose to divide HD AMS interventions, whether inpatient or outpatient, into six areas: prevention, diagnosis, treatment, education and empowerment, monitoring, and research.

## Methods and results

**Figure 1.**
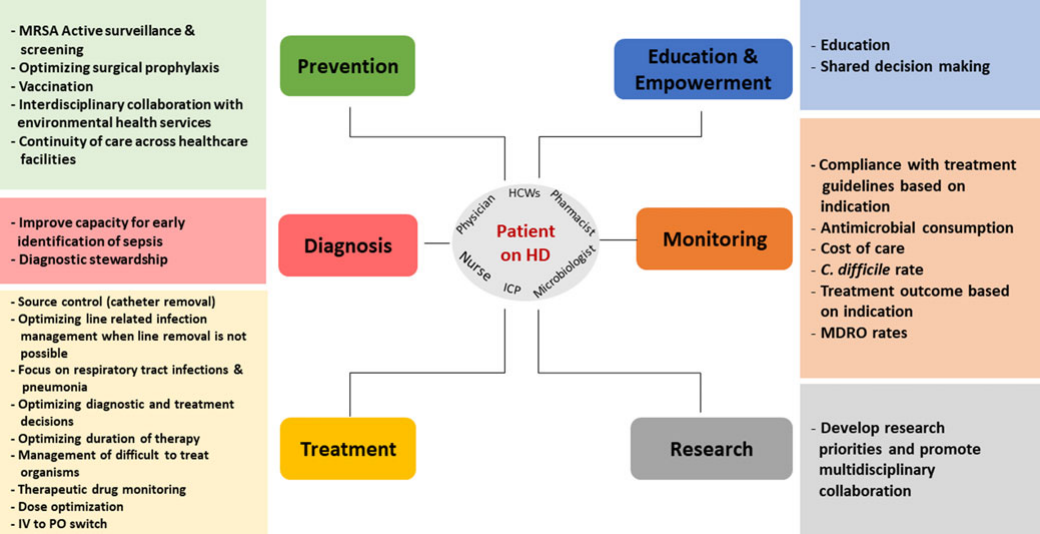
Summary of best AMS interventions in patients on intermittent hemodialysis.

Embase, PubMed, the Cochrane Library, CINAHL databases, and Web of Science were searched in a nonsystematic approach for relevant articles on AMS in the inpatient and outpatient HD setting using all synonyms for the words “antimicrobials,” “dialysis,” and “stewardship.” A total of 47 published articles were included in the review. The results are categorized into six intervention themes with their respective recommendations and are summarized in Figure 1 and Table 1.

**Table 1.**
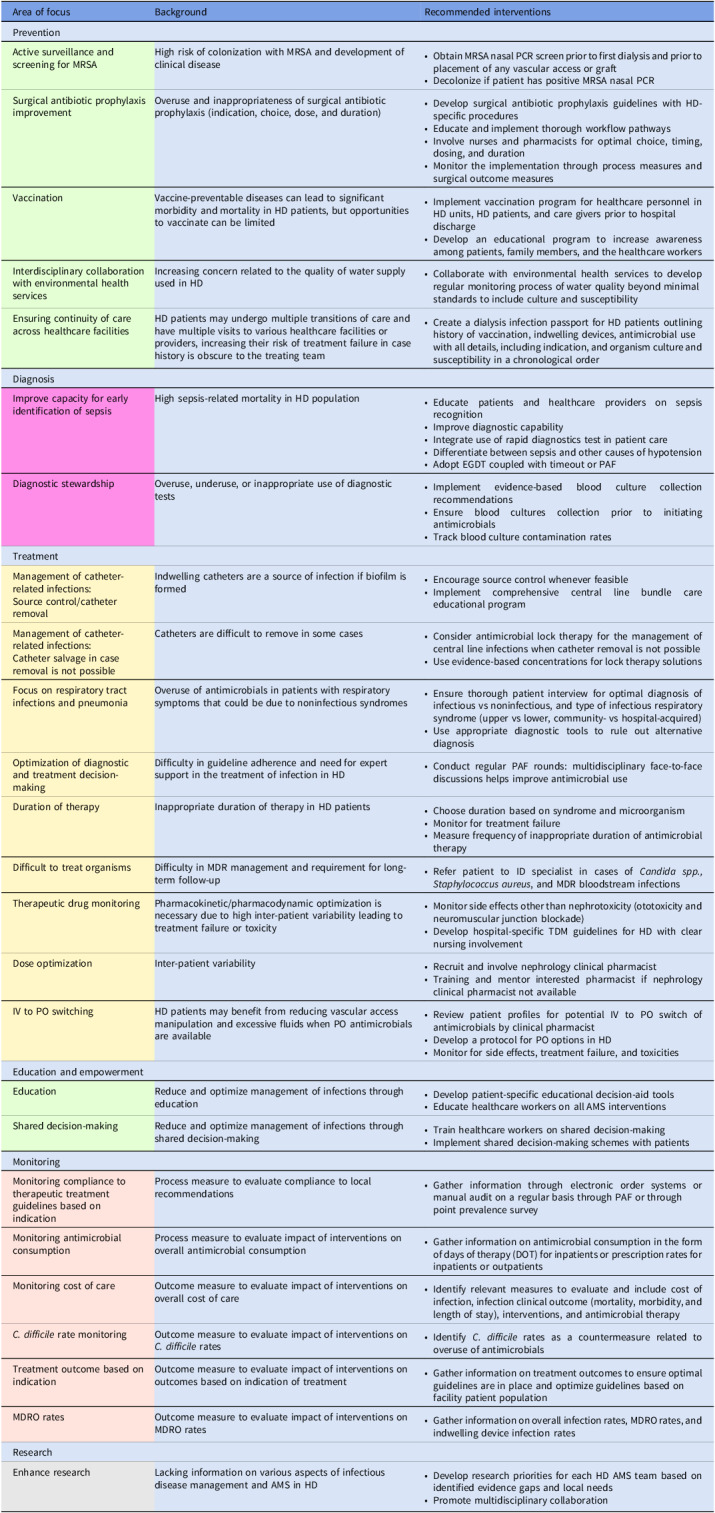
Overview of possible AMS interventions in patients on intermittent hemodialysis

### AMS success through multidisciplinary teams

Hospitals and healthcare settings differ in their coordination of HD; therefore, interventions must be tailored to the specific needs of each facility. To implement AMS in HD, it is first important to identify if an AMS program already exists. If so, the nephrologists or HD nurses can approach the AMS team to develop a task force focused on HD, or vice versa. If not, the setup of a core group that meets regularly to discuss plans, implement, and monitor outcomes is recommended. Although the mentioned interventions can be implemented without a core team, it is optimal to have a dedicated or partially dedicated core composed of an infectious disease (ID) and/or AMS physician, and ID/AMS clinical pharmacist, a microbiologist, an infection control nurse alongside a nephrologist, an HD nurse champion, and a vascular surgeon. Other specialists including a quality improvement specialist, a representative from logistics, an information technology specialist if an electronic order system and database is being used, and other members as seen fit can also be invited. In settings where these specialists are not available, a champion nurse, pharmacist, and physician could implement these initiatives with special attention to quality improvement and behavioral change incentive concepts. Leadership support is essential, and this core group should report their activities to a senior hospital leader. The multidisciplinary team will implement AMS initiatives embedded in ongoing workflows pertaining to HD patients such as developing patient counseling and education as needed, adding AMS considerations in pathways and management protocols, developing flagging through manual or electronic decision support if possible,^4,5^ and altering data collection to allow subgroup analysis of HD patients.

### AMS interventions through infection prevention

A combination of HD-specific infection prevention efforts in addition to the gold standard practice have been proposed to improve care and provide a safe environment.

First, active surveillance and screening for methicillin-resistant *Staphylococcus aureus* (MRSA) through nasal PCR is recommended in patients prior to their first HD session as well as prior to undergoing vascular access, arteriovenous (AV) fistula creation or peritoneal dialysis insertion. If the PCR is positive, decolonization must be attempted since the risk of *S. aureus* infection in HD patients is 100 times higher than other patient populations and causes around 34% of bloodstream infections.^6^ The decolonization protocol includes 5 days of twice-daily nasal mupirocin ointment and twice-daily 4% chlorhexidine body wash for 7 days. Decolonization may occur in the outpatient setting; however, this may be challenging as compliance may be low but is still possible with follow-up and patient support.^7^ Failure rates of decolonization in HD patients have been reported ranging from 16.7% (*N* = 54) to 30%–40%.^8,9^ Additionally, the risk of re-colonization is significant due to repeated exposure and concentration of risk factors within a small geographical area in the HD centers.^7^ Monitoring nasal MRSA carriage and subsequent clinical infection could be an excellent AMS initiative that generates local data on the need and frequency to decolonize patients.

Due to the higher risk of acquisition of *Candida auris* and Carbapenem-resistant *Enterobacterales* in some facilities, screening has been conducted for infection control purposes. There are no established recommendations toward these practices outside of outbreaks or special situations, likely due to the lack of successful decolonization options.^10^ More data are needed as HD patients are not commonly included in relevant studies^11^ and strict infection control measures must be followed when caring for HD patients colonized with such organisms.

Regarding surgical antibiotic prophylaxis, the only situation that requires intravenous antibiotic prophylaxis is the insertion of a peritoneal dialysis catheter^12^ where cefazolin or vancomycin may be used. The benefit of administering surgical antibiotic prophylaxis prior to other dialysis access surgeries has not been consistent.^13^ A targeted AMS intervention is to ensure optimal implementation of these practices since one of the concerns is that the indication, choice, dose, administration time, or duration of antibiotic prophylaxis in peritoneal dialysis catheter insertion may be inappropriate, or that antibiotics may be used as surgical prophylaxis without clear indication.^14,15^ Establishing a dedicated HD surgical prophylaxis improvement project can be a low-hanging AMS fruit. Furthermore, developing guidelines of when to use cefazolin and vancomycin in addition to MRSA screening and decolonization protocols are important.

Vaccination of HD patients can be effective in reducing vaccine-preventable diseases. Several studies showed that HD patients have higher risk of morbidity and mortality due to respiratory infections.^16,17^ The impaired immune response in HD can lead to an increased susceptibility to vaccine-preventable diseases and increases morbidity and mortality independently.^18^ It also increases patients’ risk of exposure to healthcare systems and antimicrobials, which increases the risk of MDR organisms. Therefore, vaccines not only reduce the risk of these diseases, but they can also reduce the risk of AMR.^19^ The best solution to improving vaccination uptake is to vaccinate patients prior to hospital discharge. In addition, education on the importance of vaccination is needed as knowledge and attitudes toward vaccinations and factors influencing vaccination adherence may vary.^20,21^

Environmental surveillance as prevention specifically tailored toward water quality in HD is an important AMS intervention. Evaluation of water used in HD continues to be a requirement, looking at the quantity of organisms in water as well as the type of organisms isolated. A study in Morocco^22^ identified that even though 51/54 tested samples passed national and European standards for final dialysate quality (heterotrophic plate counts of <100 CFU/mL, absence of coliforms in 100 mL, endotoxin level of <0.25 EU/mL), 50% of the samples collected after carbon filtration showed positive cultures, including *Pseudomonas spp* (*N* = 12), *Stenotrophomonas maltophilia* (*N* = 16), and *Burkholderia cepacia* (*N* = 4). In addition, 43.75 % were MDR and 18.75% were extensively drug-resistant. Collaboration with environmental health services to regularly monitor water quality beyond minimal standards and to include culture and susceptibility to monitor for possible water-borne bloodstream infections should become a standard.

Continuity of care is another novel approach to prevention through patient empowerment and the development of the concept of dialysis infection passport. Reports on the concept of medical passport have been documented in dialysis, in vaccination passports historically,^23^ and even in the form of a downloaded personal phone application APP.^24^ The development of a similar approach for dialysis patients with the inclusion of a dedicated section on history of vaccination, indwelling devices, antimicrobial use including indication, and organism culture and susceptibility in a chronological order can improve infection management when HD patients visit multiple healthcare facilities. This approach incorporates the knowledge that system barriers can often lead to failed therapy and that a system-based approach that acknowledges system weaknesses is needed in AMR mitigation.^25^

### AMS interventions focused on optimizing diagnosis

One of the first elements to consider in HD AMS is the need for early identification of sepsis due to high sepsis-related mortality. With in-hospital septic shock mortality rising up to 40% in HD patients in some reports,^26^ early goal-directed therapy (EGDT)^27^ is necessary to decrease sepsis-related mortality. However, there is a particular concern in HD settings where patients may have a variety of syndromes that may mimic sepsis such as hypotension, shortness of breath, and tachycardia alongside less capacity to mount a fever in the setting of lacking diagnostic tools apart from cultures and serum lactate level that can lead to misidentification of sepsis in HD patients and increasing their exposure to unnecessary antibiotics in an effort to avoid missing sepsis. The recent increase in procalcitonin availability initially spurred hope, and although a procalcitonin cutoff point of 0.75 ng/mL for identification of sepsis in HD has been suggested,^28^ most still avoid using procalcitonin as results may not be representative due to accumulation in renal failure.^29^ In an effort to avoid under-diagnosis of sepsis in HD, we recommend to increase education of all healthcare workers on recognition of sepsis and its mimics in HD patients, urgent implementation of EGDT, follow-up with strict diagnostic stewardship to improve diagnostic capacity as well as conducting 48- to 72-hour review through timeouts or prospective audit and feedback (PAF). This approach focuses on the typical AMS approach of aggressive initiation of antimicrobials followed by re-evaluation.

Diagnostic stewardship focuses on improving the use of diagnostics when needed and not more.^30^ Missing cultures, taking cultures after starting antimicrobials, inappropriate sample collection techniques, not documenting the culture indication, not obtaining central and peripheral blood cultures at the same time, and obtaining cultures unnecessarily are all examples of inadequate use of diagnostics that can lead to reduced diagnostic test yield, overuse of precious resources, and increased unnecessary treatment due to contamination of sample.^30^ False-positive blood cultures (contamination) can be as high as 50% of blood samples, although a 3% cutoff rate is recommended and <1% contamination rate should be sought.^31^ Coagulase-negative *Staphylococcus spp.* has been the most commonly isolated organism from blood culture leading prescribers to use vancomycin accounting for 40%–75% of all positive blood cultures.^32,33^ Variations in recommendations when it comes to blood culture sampling exist. In adults, if concerned about a central venous catheter (CVC)-related infection, the Infectious Disease Society of America (IDSA) guidelines recommend to obtain a set of peripheral and a set from central lines.^34^ Of note, one set of blood sampled for culture is equivalent to 20 mL drawn from the same site, 10 ml of sample would be inoculated in one aerobic and another 10 mL into an anaerobic blood culture bottle. This approach is debated and some recommended taking one set equivalent from peripheral source followed directly by another set from the dialysis machine arterial port.^35^ If CVC infection is not suspected, a set sampled from the dialysis machine arterial port followed by another set taken 20–30 minutes after the first set from a vascular access (fistula/graft).^36^ In both cases, a minimum of 40 mL of blood from two distinct sites would have been sampled, and this is the gold standard minimum quantity of blood to yield optimal culture results,^37^ a practice that is not always implemented leading to possible overcalling of what would usually be considered contamination as positive culture due to the lack of a comparator blood culture sample. The adverse impact of taking less blood volume than required is highlighted by a study that found that the odds of identifying an organism from blood cultures increases by 13% for every additional ml of drawn blood.^38^

Accordingly, an initial approach to improving diagnostic stewardship is the development of HD-specific pathways for diagnosis of infection and its management, including clear diagnostic sampling requirements for blood as well as all other diagnostic tests prioritized by the likelihood of overuse.

### AMS interventions focused on optimizing treatment of infectious diseases in HD

As one of the most common infections in HD patients in CRBSI, source control is paramount to optimal management. A retrospective study from Malaysia identified that of 496 HD patients with suspected CRBSI that were evaluated, 175 events occurred with 4.2% that were CRBSI and 4.8% that were catheter colonization.^39^ Those who grew gram-negative organisms and whose catheter was salvaged had worse outcomes (*p* = 0.026). Source control in the setting of CRBSI requires complete line removal without using a guide wire. Among 52 patients with septic arthritis in Taiwan, the presence of tunneled cuffed catheter was more common in those with positive blood cultures (41.7 vs 7.5%, *p* = 0.011) which was also a predictor of longer hospital stay (OR = 7.60; Cl 1.31–44.02; *p* = 0.024) and higher mortality (OR = 14.33; Cl 1.12–183.18; *p* = 0.041).^40^ This helps clinicians identify patients with tunneled cuffed catheter infection that are more likely to have worse outcomes and need close monitoring as well as aggressive source control and pharmacologic management. It is important to foster an environment of collaboration and communication, and this is one of the reasons why having a vascular surgeon or vascular access specialist onboard with the HD AMS team is crucial. Together, solutions for difficult to manage situations can be addressed with clarity rather than having back and forth written communication by ID/AMS requesting line removal when the team is either not aware of the treatment consequences or unable to remove the line which often leads to frustration. In case the line cannot be removed, considering the role of antibiotic lock therapy (ALT) is important.

A specific focus must be considered on viral respiratory tract infections and pneumonia in HD. A meta-analysis conducted on 42 studies enrolling 8932 patients compared COVID-19-related morbidity and mortality in patients with and without chronic kidney disease (CKD) or acute kidney injury (AKI). The study showed that those with CKD and COVID-19 have an increased risk of disease progression (OR 2.31, 95% CI 1.64–3.24) or death (OR 5.11, 95% CI 3.36–7.77).^41^ Increased mortality of HD patients in case of community or hospital-acquired respiratory tract infections has also been reported. A recent meta-analysis showed that the presence of kidney disease was associated with increased mortality in patients with respiratory tract infections, especially in pneumonia both community and hospital-acquired [RR 1.96 (95% CI 1.48–2.59)].^42^ The increased risk was attributable to the underlying cardiovascular risks as well as increased underlying systemic inflammation and oxidative stress. In parallel, lacking sensitive diagnostic tools and even low access to the available tools in low-resource settings (such as chest x-ray) affect the ability to differentiate between infection and other causes of respiratory symptoms. This leads prescribers to choose possible unnecessary antimicrobial treatment over lack of action. The clinical and policy implications are that prevention of respiratory illness through vaccination of patients, their caretakers, and their healthcare providers is necessary. In addition, the development of early diagnosis pathways with thorough patient interview in addition to respiratory diagnostic stewardship as previously mentioned is required, and finally, optimizing antibiotic empirical and targeted management of pneumonias is important.

Once the optimal antimicrobial has been determined, therapeutic drug monitoring (TDM) must be considered, especially since vancomycin and aminoglycosides are core options in HD infections, both of which require TDM. TDM not only ensures optimal levels to increase the chance of target attainment and thus improve the likelihood of success, but it also reduces the risk of other toxicities such as ototoxicity and neuromuscular junction blockade.^43^ The role of AMS is to ensure that every single patient on these antibiotics is evaluated for the need for TDM and is managed by a pharmacist or clinical pharmacist. It is necessary to highlight that evaluation of the need for therapy continuation and subsequent discussion with the treating physician is within the role of the TDM pharmacist.

Throughout the process of developing an HD AMS, compliance with recommendations and continuous education is needed to foster collaboration between the teams and optimize diagnostic and treatment decision-making. Antibiotic use in HD can be inappropriate in 30%–37% of cases,^44,45^ and even if initially appropriate, duration of therapy and lack of targeted therapy can be inappropriate. This is why PAF is a necessary tool for AMS. PAF can be accomplished through regular face-to-face multidisciplinary meetings including all the AMS core team members. The frequency can be determined based on patient load and availability of participants. Case-based discussions of patients on antimicrobials with clearly defined recommendations and documentation during rounds can help improve the overall antimicrobial use process and facilitate data collection relating to monitoring. During these meetings, duration of therapy, formal ID referral for difficult to treat organisms, dose optimization, and IV to PO switch can be discussed. The meetings must be consistent, and the performance should be discussed with all members of the core team and the extended relevant healthcare workers, and finally, with hospital leadership.

A recurring theme in AMS HD is the decision about diagnosis and the decision about management. The diagnosis is usually agreed on by the treating physician, but optimal management is often also left for the treating physician. The role of a nephrology clinical pharmacist that can collaborate with the treating team and the AMS team is of crucial importance for most of the interventions already discussed. The role of nephrology clinical pharmacists has been recurrently established in the literature with dedicated standards of clinical practice published in 2013.^46^ Various types of interventions have been documented varying from improvement of dosing, identification and management of drug interactions and adverse events, medication reconciliation, and significant cost reductions confirmed.^47,48^

### AMS interventions focused on education and patient empowerment

A patient-centered care approach is recommended for all AMS initiatives. Studies have shown better outcomes when using shared decision-making related to vascular access which may then be translated to better infection risks.^49,50^ Patients reported low understanding of the complications of catheter use and the process of AV access placement, and it is suggestion that patient-friendly decision aid tools to be formulated to improve patient expectations and increase active participation in decision-making and thus better compliance to recommendations as well as general fostering better patient–healthcare worker relationships and improving patient wellbeing. In addition, healthcare worker education is also necessary throughout the implementation of all interventions, although it is not the mainstay of intervention implementation.

### AMS interventions focused on monitoring process and outcome measures

Implementation of quality and safety improvement initiatives such as AMS requires evaluation and follow-up. This can be accomplished through the development of a performance indicator monitoring process. In the case of AMS in HD, we recommend monitoring compliance to therapeutic treatment guidelines based on indication, antimicrobial consumption, cost of care, *C. difficile* rate, treatment outcome based on indication, and MDR organisms’ rates (Table 1).

### AMS interventions focused on AMS HD research

A final element of HD AMS is the generation of new data to help improve decision-making in this field. HD patients tend to be excluded from clinical research. With the high mortality rates related to infections, more information is urgently needed to support evidence-based cost-effective interventions. A recent example of the gaps that require focused research related to risk factors of worse outcomes in HD was described by the American Center of Disease and Control report that associated race with worse infection outcomes in Hispanic patients with a 1.4 adjusted rate ratio compared to non-Hispanic White patients.^6^ This outcome was linked to poverty, lower education, and crowding. We refer back to the World Health Organization research priority agenda^2^ which highlights the importance of research in vulnerable patient populations. The impact of social determinants of health on HD outcomes can affect patients anywhere in the world, and more data are needed on this specific issue. HD teams are required to breakdown demographic data when monitoring patient outcomes. Other research priority topics include effective preventive strategies for CRBSI, ALT solution, optimal timing of antimicrobials dosing during HD, optimal method to administer and monitor vancomycin and aminoglycosides,^51^ prevention of *Staphylococcus spp*. infections through vaccination,^30^ and decolonization,^7^ optimal frequency of vaccination in HD patients who do not achieve sero-responsiveness, improvement of vaccine-induced sero-responsiveness and evaluation of vaccination rates in HD patients, and vaccine-preventable diseases.

## Conclusion

As more facilities develop and expand their AMS program, attention to the HD population can help mitigate existing infectious-related and AMR risk factors in this group and improve clinical outcomes. Interventions must be tailored by multidisciplinary teams to the specific needs of each facility and based on a thorough evaluation of the AMR, infection rates, and prescribing practices. Further research is needed on the impact of dedicated AMS initiatives and optimal interventions that can improve care of HD patients.
